# Neural Representations of Observed Interpersonal Synchrony/Asynchrony in the Social Perception Network

**DOI:** 10.1523/JNEUROSCI.2009-22.2024

**Published:** 2024-03-25

**Authors:** Maria Tsantani, Daniel Yon, Richard Cook

**Affiliations:** ^1^Department of Psychological Sciences, Birkbeck, University of London, London WC1E 7HX, United Kingdom; ^2^School of Psychology, University of Leeds, Leeds LS2 9JU, United Kingdom; ^3^Department of Psychology, University of York, York YO10 5DD, United Kingdom

**Keywords:** extrastriate body area, interpersonal synchrony, social interactions, social perception, superior temporal sulcus

## Abstract

The visual perception of individuals is thought to be mediated by a network of regions in the occipitotemporal cortex that supports specialized processing of faces, bodies, and actions. In comparison, we know relatively little about the neural mechanisms that support the perception of multiple individuals and the interactions between them. The present study sought to elucidate the visual processing of social interactions by identifying which regions of the social perception network represent interpersonal synchrony. In an fMRI study with 32 human participants (26 female, 6 male), we used multivoxel pattern analysis to investigate whether activity in face-selective, body-selective, and interaction-sensitive regions across the social perception network supports the decoding of synchronous versus asynchronous head-nodding and head-shaking. Several regions were found to support significant decoding of synchrony/asynchrony, including extrastriate body area (EBA), face-selective and interaction-sensitive mid/posterior right superior temporal sulcus, and occipital face area. We also saw robust cross-classification across actions in the EBA, suggestive of movement-invariant representations of synchrony/asynchrony. Exploratory whole-brain analyses also identified a region of the right fusiform cortex that responded more strongly to synchronous than to asynchronous motion. Critically, perceiving interpersonal synchrony/asynchrony requires the simultaneous extraction and integration of dynamic information from more than one person. Hence, the representation of synchrony/asynchrony cannot be attributed to augmented or additive processing of individual actors. Our findings therefore provide important new evidence that social interactions recruit dedicated visual processing within the social perception network that extends beyond that engaged by the faces and bodies of the constituent individuals.

## Significance Statement

The presence of interpersonal synchrony is a critical cue when appraising the nature and content of social interactions from third-person perspectives. However, little is known about its representation within the human visual system. Here, we use fMRI to reveal distributed representations of interpersonal synchrony/asynchrony in several regions of the social perception network, notably extrastriate body area and superior temporal sulcus. There is growing speculation that the perception of social interactions engages specialized visual processing beyond that recruited by the faces and bodies of the constituent individuals. Critically, perceiving interpersonal synchrony requires the simultaneous extraction and integration of dynamic information from more than one person. These results therefore provide key new evidence of dedicated multiactor processing within the social perception network.

## Introduction

The visual perception of individuals has been an active area of research for many years. This research tradition has revealed dedicated neural substrates for the visual processing of faces (Haxby et al., 2000; Duchaine and Yovel, 2015), bodies (Peelen and Downing, 2007), and actions (Blake and Shiffrar, 2007). In comparison, we know relatively little about the neural mechanisms that support the perception of multiple individuals and the interactions between them. Over the last decade, however, studies using functional magnetic resonance imaging (fMRI) have consistently implicated two regions of the social perception network in the visual processing of social interactions.

Initial findings suggest that the extrastriate body area (EBA; Abassi and Papeo, 2020, 2022) and posterior superior temporal sulcus (pSTS; Kujala et al., 2012) show greater activation when participants view static images of face-to-face dyads, than when they view images of back-to-back dyads. Similar findings have been observed with dynamic stimuli; for example, authors have described stronger responses in EBA (Bellot et al., 2021; Landsiedel et al., 2022) and pSTS (Centelles et al., 2011; Isik et al., 2017; Walbrin et al., 2018; Bellot et al., 2021; Landsiedel et al., 2022) when participants view interacting dyads (i.e., two actors shown facing each other, performing contingent, related actions) than noninteracting dyads (i.e., two actors shown nonfacing and performing unrelated actions).

Neural representations in EBA and pSTS may encode information about the nature and content of social interactions. For example, stronger univariate responses are seen in both EBA and pSTS when participants view static images of semantically incongruous interactions, than when viewing images of congruous interactions (Quadflieg et al., 2015). Having employed multivoxel pattern analysis (MVPA), Walbrin and Koldewyn (2019) found that a classifier trained on pSTS and EBA responses was able to discriminate different types of social interaction (arguing vs celebrating vs laughing). The portion of pSTS sensitive to social interactions also discriminates different types of interactions between moving abstract shapes (Isik et al., 2017; Walbrin et al., 2018). For example, Isik et al. (2017) found that a classifier trained on responses in pSTS was able to discriminate animations depicting helping versus hindering actions.

The present study sought to further elucidate the visual processing of social interactions by identifying which regions of the social perception network represent interpersonal synchrony. Interpersonal synchrony refers to the temporospatial alignment of movements between interacting individuals that often occurs automatically, effortlessly, and unintentionally (Marsh et al., 2009; Hoehl et al., 2021). Perceived synchrony strongly influences our interpretation of dynamic social scenes. For example, dyads moving in synchrony are more likely to be perceived as a social unit than those moving asynchronously (Lakens, 2010; Lakens and Stel, 2011), and synchrony affords attributions of rapport (Miles et al., 2009; Lakens and Stel, 2011) and affiliation (Latif et al., 2014). At present, however, little is known about the neural representation of interpersonal synchrony within the visual system (Cracco et al., 2022).

We used MVPA to investigate whether activity in face-selective, body-selective, and interaction-sensitive regions across the social perception network supports the decoding of synchronous versus asynchronous head movements. We manipulated the presence of interpersonal synchrony between the actors while holding the basic dyadic arrangement (i.e., face-to-face) constant. We predicted that responses in EBA and pSTS, which have been consistently implicated in the visual processing of social interactions (Centelles et al., 2011; Kujala et al., 2012; Isik et al., 2017; Walbrin et al., 2018; Walbrin and Koldewyn, 2019; Abassi and Papeo, 2020, 2022; Bellot et al., 2021), would be sensitive to interpersonal synchrony.

## Materials and Methods

### Participants

Thirty-three healthy right-handed adults aged 18–50 participated in the study. Our sample size was based on similar fMRI studies within the field (Abassi and Papeo, 2022). Participants reported normal or corrected-to-normal vision. One participant was excluded because of an incomplete dataset. The final sample consisted of 32 participants (26 female, 6 male) with a mean age of 27.66 years (SD = 6.93; range, 18–43). The study was approved by the relevant ethics committees of Birkbeck, University of London, and University College London. All participants provided written informed consent.

### Stimuli

**Figure 1.**
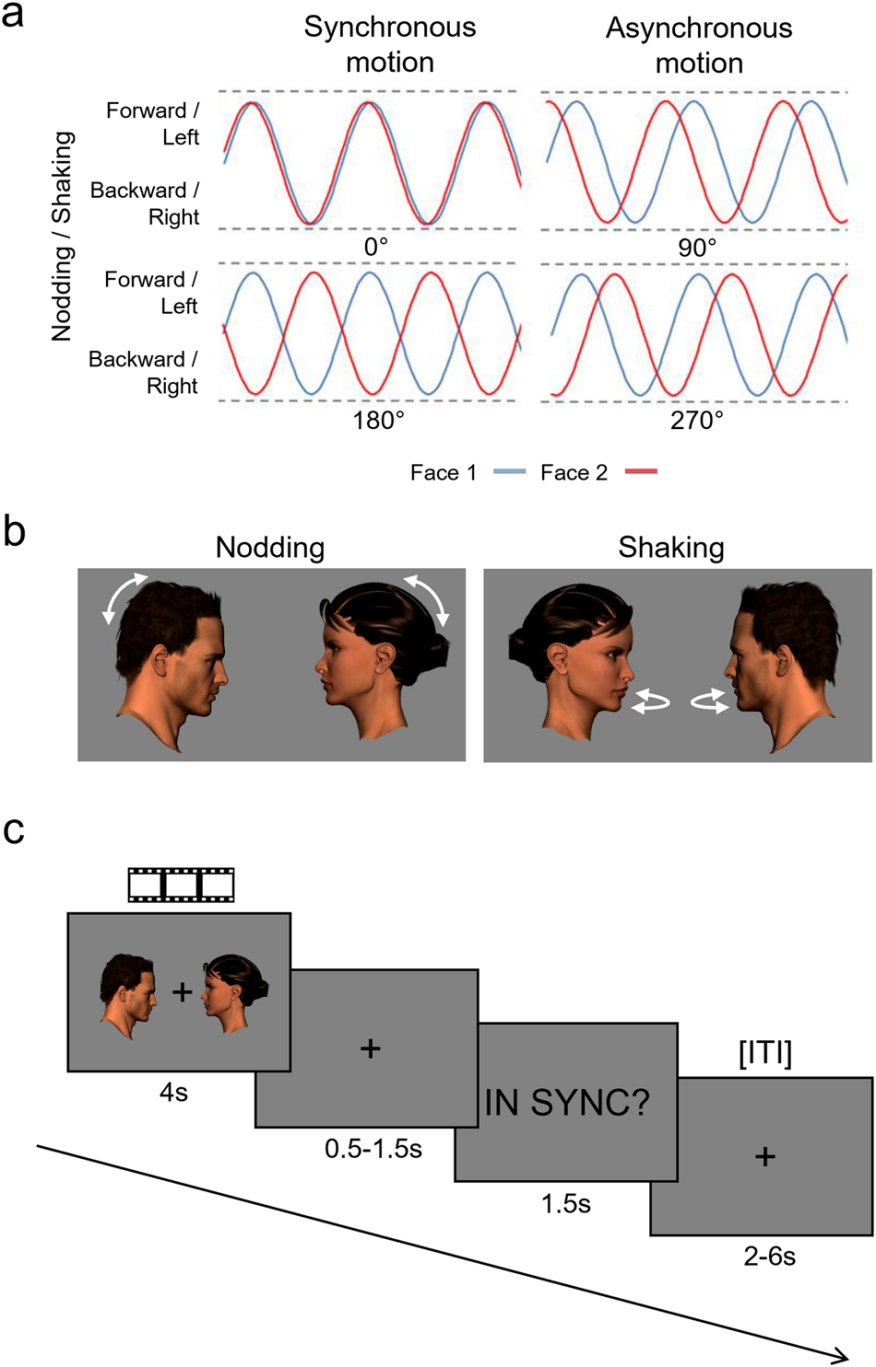
***a***, The relative-phase relationships between Face 1 and Face 2 in synchronous stimulus videos (0° and 180° phase offset) and asynchronous stimulus videos (90° and 270° phase offset). ***b***, Illustration of nodding and shaking movements in the stimulus videos. ***c***, An example trial of the main experiment (stimulus images and text are magnified for illustration purposes). After viewing each video, participants responded with a yes/no button press to the question “in sync?” or “out of sync?”. ITI, intertrial interval.

The video stimuli featured a pair of avatar heads (one male, one female) that moved synchronously or asynchronously relative to each other. Each three-dimensional model head was generated and rendered in Poser Pro 11.2 (Bondware). The two heads were shown in profile view facing each other (Fig. 1*b*). For each model, we created a sequence of 40 images showing the head progressively moving forward and back (“nodding”) and a sequence of 40 images showing the head progressively rotating from left to right (“shaking”). The images were used to create two types of synchronous videos featuring 0° (in-phase synchrony) and 180° (antiphase synchrony) relative-phase offsets and two types of asynchronous videos featuring 90° and 270° relative-phase offsets (Fig. 1*a*).

The order of the images for the head on the left was always the same, whereas the starting point in the sequence of images for the head on the right was modified to create the different phase offset conditions. For the 0° phase offset, the movement of the two heads was mirrored. For the 180° phase offset, the position of the heads was at opposite points of the movement cycle (e.g., in the nodding stimuli, one head would move forwards while the other moved backward). For the 90° phase offset, the right head led the left head by a quarter of a cycle, and for the 270° phase offset, the right head led the left head by three quarters of a cycle.

Videos of nodding and shaking movements were compiled using MATLAB (version R2019b). To avoid movement cycle time locking to the TR of the MRI scanner, we made two sets of videos with different frame rates (28 fps and 32 fps). All videos were 4 s long. The 28 fps videos depicted 2.8 cycles, and the 32 fps videos depicted 3.2 cycles. Two versions of each stimulus were produced, one with the female head on the left and one with the female head on the right. In total, there were 32 video stimuli: two movement types (nodding, shaking) x four phase offsets (0°, 180°, 90°, 270°) x two frame rates (28 fps, 32 fps) x two model configurations (female left, female right).

### MRI data acquisition

Participants were scanned using a 3.0 Tesla Siemens Prisma MRI scanner with a 32-channel head coil. Scanning took place at the Birkbeck-UCL Centre for Neuroimaging. We acquired whole-brain T1-weighted anatomic scans using MPRAGE (1.0 mm isotropic; 208 sagittal interleaved slices; PAT, factor 2; PAT mode, GRAPPA; TR, 2,300 ms; TE, 2.98 ms; flip angle, 9°; matrix, 256 × 256; FOV, 256 mm). For the functional runs, we acquired T2*-weighted functional scans using EPI (3.0 mm isotropic; PAT, factor 2; PAT mode, GRAPPA; 34 ascending sequential slices; TR, 2,000 ms; TE, 30 ms; flip angle, 78°; matrix, 64 × 64; FOV, 192 mm). Slices were positioned at an oblique angle to include the temporal, occipital, and frontal lobes and as much of the parietal lobe as possible.

### Experimental design and statistical analysis

#### Design of the main experiment

The experimental runs presented videos of pairs of heads moving synchronously or asynchronously in an event-related design. The experiment was presented using the Psychophysics Toolbox v3.0 (Brainard, 1997; Pelli, 1997) in MATLAB (version 2014a) and was projected to a screen (1,920 × 1,200 pixels, 28.8 × 18 cm, 60 Hz) at the back of the scanner bore, which the participants viewed through a mirror attached to the head coil. Participants viewed the screen from a distance of approximately 55 cm (±3 cm) and were instructed to keep their gaze focused on the central fixation cross for the duration of the experiment.

There were 32 trials in each run. Each video stimulus was presented once per run without repetition. Each trial began with a stimulus video (4 s), followed by a fixation screen with a jittered duration of between 0.5 and 1.5 s (averaging 1 s; Fig. 1*c*). Participants then viewed a response screen with the question “in sync?” or “out of sync?” and had 1.5 s to respond “yes” or “no” using buttons on a handheld button box. The response screen was followed by a jittered intertrial interval of between 2 and 6 s (averaging 4 s) featuring a fixation screen. A fixation cross was present in the center of the display at all times except during the response screen. The stimuli were presented in a random order. The pairing of the stimuli with the two versions of the task question (in/out of sync) was pseudorandomized. The randomization was constrained such that the 28 fps and 32 fps versions of each stimulus appeared with a different question. At the end of the run, participants received feedback on their task performance (proportion correct). To familiarize them with the experimental task, participants first performed a practice run in which they received feedback (correct/incorrect) after each trial. Participants completed eight runs of the experiment lasting ∼6 min each.

#### Functional localizers

We used a standard localizer for face- and body-selective regions—described in detail by Pitcher et al. (2011)—with permission from the authors. In a blocked design, participants viewed videos of moving faces, moving body parts (excluding the face), and moving objects, while performing a one-back task. The 3 s videos were presented randomly in 18 s blocks. Participants completed two runs of the 12 stimulus blocks (4 blocks per stimulus category), presented in a pseudorandom order that prevented the consecutive presentation of blocks featuring stimuli from the same category. Participants also viewed three 18 s fixation blocks situated at the start, middle, and end of each run. The duration of each run was 276 s (4.6 min). Across both runs, participants viewed eight blocks of faces, eight blocks of bodies, and eight blocks of objects. All participants completed the face/body localizer after the main experiment runs.

We used a second functional localizer (Isik et al., 2017; Walbrin et al., 2018) to identify the interaction-sensitive region of pSTS (STS-I), with permission from the authors. In a blocked design, participants passively viewed videos of moving point-light displays of two individuals who were either interacting or performing independent actions. Videos of varying lengths (between 3 and 8 s) were grouped together and presented in 16 s blocks. Participants completed two runs consisting of 12 stimulus blocks (6 per condition) and 3 fixation blocks situated at the start, middle, and end of each run. The presentation of the stimulus blocks alternated between the two stimulus categories and always began with an interactions block in Run 1 and an independent actions block in Run 2. The predefined stimulus groupings presented in each block were randomized across runs. The duration of each run was 246 s (4.1 min). Across both runs, participants viewed 12 blocks of interactions and 12 blocks of independent actions. All participants completed the STS-I localizer after the face/body localizer.

#### Data preprocessing and modeling

Functional images were preprocessed and analyzed using Statistical Parametric Mapping (SPM12; Wellcome Department of Imaging Science; www.fil.ion.ucl.ac.uk/spm) in MATLAB (version R2021a). The first five (for the main experiment runs) or three (for the functional localizers) EPI images in each run served as dummy scans and were discarded before preprocessing to allow for T1 equilibration effects. Images within each brain volume were slice-time corrected using the middle slice as a reference and were then realigned to correct for head movements using the first image as a reference. Functional images from the main experimental runs were not smoothed, whereas images from the localizer runs were smoothed with a 6 mm Gaussian kernel (full width at half maximum). The participants’ structural image in native space was coregistered to the realigned mean functional image and was segmented into gray matter, white matter, and cerebrospinal fluid, saving the forward and inverse deformation fields (used for transformations to MNI space from the subject's native space and vice versa).

Mass univariate general linear models (GLMs) were fitted to data from the main experiment runs, from the face/body localizer, and from the interactions localizer separately. GLMs included a regressor for the time course of each experimental condition and six nuisance regressors for estimated head motion parameters. For the main experiment runs, we also included the response screen as a regressor of no interest. Regressors modeled the BOLD response following the onset of the stimuli and were convolved with a canonical hemodynamic response function. We used a high-pass filter cutoff of 128 s and an autoregressive AR(1) model to account for serial correlations. The GLM analyses produced β images for each condition showing the associated signal change across each voxel in the brain.

#### ROI classification analyses

Classification analyses were performed using The Decoding Toolbox (TDT; Hebart et al., 2015) in MATLAB (version R2021a) at the individual-subject level. In each analysis, β images associated with two conditions of interest were used as input to a linear support vector machine classifier (LIBSVM; Chang and Lin, 2011). For all analyses, each condition was represented by eight β images, one from each run. We used a leave-one-out cross-validation procedure, whereby the classifier was trained to discriminate the two conditions based on images from seven runs, and the resulting linear discriminant function was tested on images from the remaining run, in eight cross-validation folds. For each ROI, we obtained the average classification accuracy across all folds. To determine whether classification accuracy in a given ROI was significantly greater than chance, we subjected participant accuracies to a one-sample *t* test. Near identical results were obtained using a permutation testing approach more suitable for inferences of information prevalence (Stelzer et al., 2013).

In our main analysis, we tested the decoding of synchronous versus asynchronous movement collapsing across all other stimulus characteristics, including the type of head movement (nodding, shaking). To test whether representations of synchrony and asynchrony generalize across movement types, we performed a cross-classification analysis by training the classifier to distinguish synchronous from asynchronous shaking and testing it on nodding, and vice versa. The cross-classification analysis followed the same leave-one-out procedure described above. All reported *p*-values are two-tailed. For each analysis, correction for multiple comparisons across 10 ROIs was performed using the false discovery rate (FDR) with *q* = 0.05.

#### Whole-brain searchlight classification analysis

We performed an exploratory whole-brain searchlight classification analysis to identify potential regions outside of our ROIs that discriminate between synchronous and asynchronous motion. For each participant, *β* values associated with synchronous and asynchronous motion were extracted from 6-mm-radius spheres centered on each voxel within a mask of their brain obtained from the GLM and were used as input to the classifier. The cross-validation procedure was the same as in our ROI analysis. For each participant, we obtained a brain map with classification accuracy at each voxel. Brain maps were normalized to standard MNI space using the forward deformation fields obtained during the segmentation procedure and were smoothed with a 3 mm Gaussian kernel (full width at half maximum) to account for anatomical variability across subjects. For the group-level analysis, brain maps were subjected to a one-sample *t* test to identify voxels with classification accuracies that were significantly greater than chance. The resulting *t*-contrast image was thresholded at *p* < 0.001 at the voxel level and corrected for multiple comparisons using FWE at *p* < 0.05 at the cluster level. We report the coordinates of peak voxels in MNI space and anatomical labels based on the automated anatomical labeling (AAL) atlas (Rolls et al., 2020).

#### Whole-brain univariate analyses

For these analyses only, images from the main experiment runs were normalized to standard MNI space and smoothed with a 6 mm Gaussian kernel (full width at half maximum) to account for anatomical variability across subjects. The normalized and smoothed images were subjected to mass univariate GLMs, and we defined the contrasts synchronous > asynchronous and asynchronous > synchronous. For group-level analysis, the contrast images were subjected to one-sample *t* tests. The resulting *t*-contrast images were thresholded at *p* < 0.001 at the voxel level and corrected for multiple comparisons using FWE at *p* < 0.05 at the cluster level. We report clusters with two or more voxels, coordinates of peak voxels in MNI space, and anatomical labels based on the AAL atlas (Rolls et al., 2020).

### Definition of functional ROIs

Subject-specific functional ROIs were defined using a group-constrained subject-specific method (Fedorenko et al., 2010; Julian et al., 2012), in which group-level parcels are used to constrain functionally localized ROIs in individual subjects. This method has the benefit of reducing experimenter bias in the definition of ROIs while allowing for anatomical variability in ROI locations across participants. Group-level parcels of face-selective and body-selective regions were derived in a study by Julian et al. (2012), who used the same face/body localizer as the present study and were downloaded from http://web.mit.edu/bcs/nklab/GSS.shtml. There was no available group-level parcel for the interaction-sensitive STS region (STS-I), so we derived the parcel from a group analysis of interaction localizer data from our own participant sample. To conduct a group-level analysis for the contrast interactions > independent actions, we first normalized all scans to the standard MNI template and repeated the GLM analysis for each subject. First-level *t*-contrast images were then subjected to a second-level one-sample *t* test. A parcel including the right posterior and mid-STS was created from the continuous significant voxels around the peak voxel in the resulting second-level *t*-map, thresholded at *p* < 0.001 uncorrected.

All group-level parcels in standard MNI space were transformed to the native space of each subject using the inverse deformation fields obtained during the segmentation procedure. The images were resliced to the same resolution as the functional images and trimmed to remove any voxels that were not present in the participant's brain mask obtained from the relevant GLM analysis.

Using the data from the face/body localizer, we defined face-selective ROIs by intersecting *t*-contrast images for faces > objects with group-level parcels of the left and right FFA, OFA, and STS face region (STS-F). We defined body-selective ROIs by intersecting *t*-contrast images for bodies > objects with parcels of the left and right EBA. From the interactions localizer data, we defined the interaction-sensitive STS ROI (STS-I) by intersecting *t*-contrast images for interactions > independent actions with the group-level STS-I parcel.

**Figure 2.**
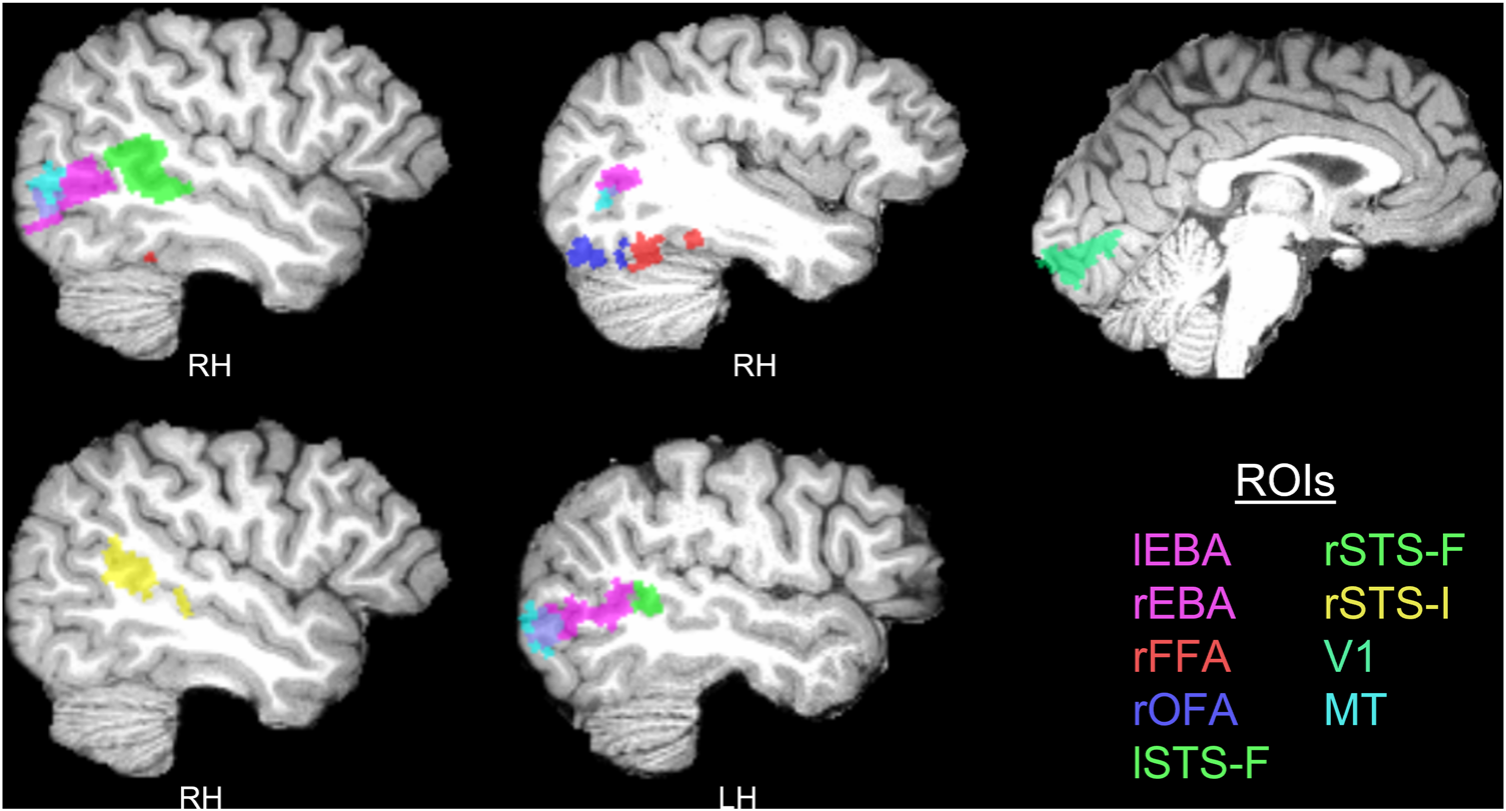
Location of ROIs in one example participant. RH, right hemisphere; LH, left hemisphere.

For each subject, ROIs were defined as the most active 30% of voxels within each group parcel. ROIs with fewer than 30 voxels were discarded. Face-selective ROIs included the rFFA (28 participants; mean vox., 55.07; range, 41–67), the rOFA (26 participants; mean vox., 44.73; range, 33–60), the rSTS-F (all participants; mean vox., 165.38; range, 129–208), and the lSTS-F (all participants; mean vox., 55.17; range, 43–70). The lFFA and lOFA could not be localized with at least 30 voxels in the majority of participants and were therefore excluded from the analysis. Body-selective ROIs included rEBA (all participants; mean vox., 146.81; range, 115–185) and lEBA (all participants; mean vox., 136.13; range, 108–171). The STS-I was localized in all participants and had an average size of 78.63 voxels (range, 45–102). The STS ROIs covered the posterior part of the STS, extending to the mid-STS in some participants. Due to a large number of overlapping voxels (*M* = 35.59, SD = 15.17; range, 7–63) between the STS-I and the larger rSTS-F ROI, we made an additional face-selective rSTS ROI that excluded all voxels that overlapped with the STS-I. This rSTS-F* ROI had an average size of 129.78 voxels (range, 98–178). The location of the various ROIs in an example participant is illustrated in Figure 2.

### Definition of nonsocial comparison ROIs

The primary focus of our classification analyses was the social perception network. However, we also examined the distributed responses seen in two nonsocial comparison ROIs: V1 and the middle temporal area (MT). V1 was selected as a control region. Given that the low-level features of our synchronous and asynchronous stimuli were closely matched, we reasoned that the distributed responses seen in V1 would be unlikely to support synchrony/asynchrony classification (Caplovitz et al., 2008). At the outset, we had no strong expectation about the ability of MT responses to support the decoding of synchrony/asynchrony. However, MT is known to play a key role in visual motion processing (Born and Bradley, 2005; Kolster et al., 2010) and has been implicated in perceptual grouping based on motion cues (Ferber et al., 2003). Moreover, there is some suggestion that MT is sensitive to visual features of dyadic interactions (Quadflieg et al., 2015; Landsiedel et al., 2022).

Probabilistic masks of V1 and MT derived by Wang et al. (2015) were downloaded from https://scholar.princeton.edu/napl/resources. Our V1 and MT masks were bilateral, and the V1 mask included ventral and dorsal subregions. Masks were transformed into each subject's native space in the same way as the functional ROIs (see above). Individual-subject V1 ROIs were defined as the 200 voxels with the highest probabilities. MT ROIs were defined as the 100 voxels with the highest probabilities.

### Data and code accessibility

β images obtained from the GLMs, task accuracy scores, and MATLAB analysis scripts are available via the Open Science Framework (https://osf.io/s6phj/?view_only=ad89001d587b4cfda4a3b286ce023332).

## Results

### Behavioural task accuracy

Participants correctly identified the stimuli as synchronous or asynchronous on 89.59% of trials on average (SD = 9.63; range, 65.23–99.61). There were no differences in accuracy between synchronous (*M* = 91.04%, SD = 9.36) and asynchronous stimulus trials (*M* = 88.13%, SD = 12.84; *t*_(31)_ = 1.418, *p* = 0.166). Accuracy was slightly higher for shaking (*M* = 90.82%, SD = 8.20) compared with nodding stimuli (*M* = 88.35%, SD = 11.71; *t*_(31)_ = 2.265, *p* = 0.031).

### ROI analyses

**Figure 3.**
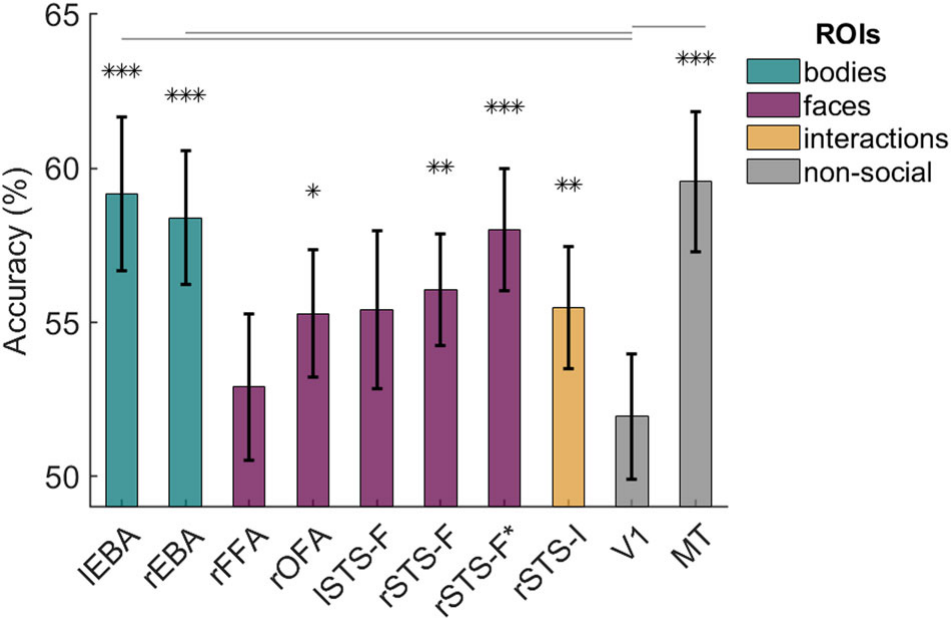
Mean classification accuracies for decoding of synchronous versus asynchronous movement in body-selective ROIs (green), face-selective ROIs (purple), an interaction-sensitive ROI (yellow), and nonsocial comparison ROIs (gray). Error bars show standard error. The asterisks indicate above-chance accuracy after FDR correction for 10 comparisons: *p ≤ 0.05; **p ≤ 0.01, ***p ≤ 0.001. The horizontal lines above the bars indicate significant pairwise differences between each comparison ROI and all other ROIs.

We found above-chance decoding of synchronous versus asynchronous movement in bilateral EBA, in rOFA, in face-selective and interaction-sensitive regions of rSTS (STS-F, STS-F*, STS-I), and in MT (Fig. 3; Table 1). The same patterns were obtained using an alternative permutation testing approach (Stelzer et al., 2013), except that the lSTS-F region was found to contain significant information after FDR correction for multiple comparisons. Classification accuracies were significantly lower in the V1 comparison region compared with lEBA (*t*_(31)_ = 3.143, *p* = 0.004), rEBA (*t*_(31)_ = 2.755, *p* = 0.010), and MT (*t*_(31)_ = 3.040, *p* = 0.005). The difference between accuracies in V1 and rSTS-F* did not survive FDR correction (*t*_(31)_ = 2.111, *p* = 0.043), and no other differences were significant (all *p*s > 0.07). There were no significant pairwise differences in classification accuracy between regions that showed significant decoding of synchronous versus asynchronous movement (all *p*s > 0.12).

**Table 1.** Mean classification accuracies for decoding of synchronous versus asynchronous movement and one-sample *t* test results

ROI	Mean accuracy (%)	Df	*t*-value	*p*-value
lEBA	59.18	31	3.681	**0.001**
rEBA	58.40	31	3.876	**0.001**
rFFA	52.90	27	1.223	0.232
rOFA	55.29	25	2.553	**0.017**
lSTS-F	55.42	29	2.120	0.043
rSTS-F	56.05	31	3.350	**0.002**
rSTS-F*	58.01	31	4.067	**<0.001**
rSTS-I	55.47	31	2.763	**0.010**
V1	51.95	31	0.961	0.344
MT	59.57	31	4.198	**<0.001**

Those *p*-values in bold survived FDR correction.

In general, the decoding of interpersonal synchrony was high in the MT comparison region, and decoding accuracy did not differ significantly between MT and the ROIs thought to be part of a distinctive social perception network (all *p*s > 0.06). One potential reason for such high decoding of interpersonal synchrony in MT is that, across participants, this ROI contained several voxels that overlapped with EBA (lEBA overlap: *M* = 22.03, SD = 8.97; range, 3–35 voxels; rEBA overlap: *M* = 28.66, SD = 14.92; range, 5–58 voxels). When overlapping voxels were excluded from each ROI, the decoding of interpersonal synchrony remained high in EBA (lEBA: *M* = 58.20%, *t*_(31)_ = 3.824, *p* < 0.001; rEBA: *M* = 60.74%, *t*_(31)_ = 5.016, *p* < 0.001). In contrast, decoding in MT dropped substantially to 53.52%, and was no longer significantly different from chance (*t*_(31)_ = 1.579, *p* = 0.125).

It is possible that chance levels of decoding seen in MT following the removal of the overlapping EBA voxels reflect the relatively small number of residual MT voxels. With overlapping voxels removed, on average, MT size was reduced by ∼50% to 49.31 voxels (range, 25–83). For comparison, lEBA size was reduced by ∼16% to 114.09 voxels (range, 92–154), and rEBA size was reduced by ∼20% to 118.16 voxels (range, 73–162). However, we note that classification accuracy remained significantly above chance in rEBA (*M* = 56.45, *t*_(31)_ = 3.029, *p* = 0.005) and in lEBA (*M* = 56.45, *t*_(31)_ = 3.083, *p* = 0.004) when the size of these ROIs was constrained to the 50 most body-selective voxels.

#### Generalization across movement type

**Figure 4.**
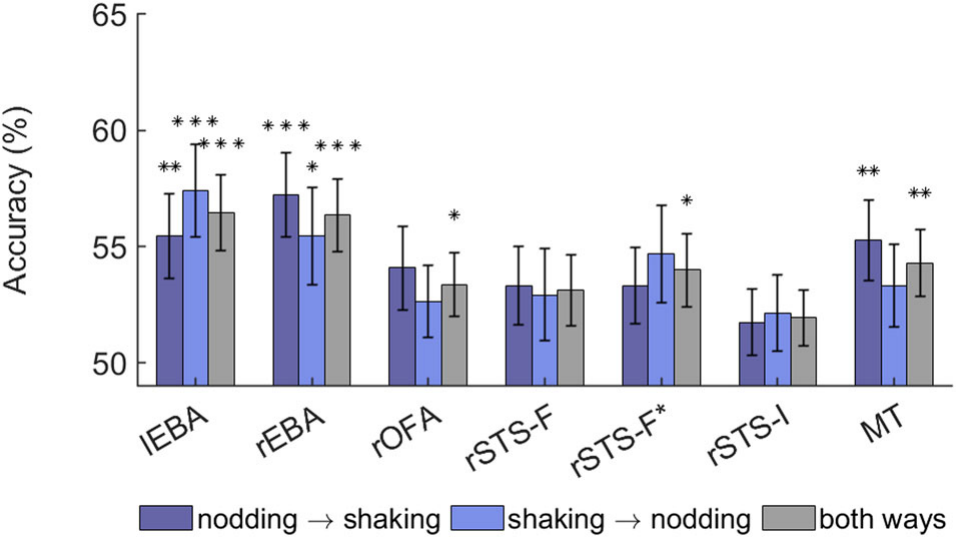
Mean cross-classification accuracies for decoding of synchronous versus asynchronous movement with generalization from nodding to shaking, generalization from shaking to nodding, and generalization in both directions. Error bars show standard error. The asterisks indicate above-chance accuracy after FDR correction for 10 comparisons: *p ≤ 0.05; **p ≤ 0.01, ***p ≤ 0.001.

Where regions showed above-chance classification in the foregoing analyses, we also examined whether representations of interpersonal synchrony generalize across nodding and shaking movement using cross-classification (Fig. 4). We found significant above-chance cross-classification of synchronous versus asynchronous movement across both directions (generalizing from nodding to shaking and from shaking to nodding) in lEBA (N→S: *M* = 55.47%, *t*_(31)_ = 3.016, *p* = 0.005; S→N: *M* = 57.42%, *t*_(31)_ = 3.724, *p* = 0.001) and rEBA (N→S: *M* = 57.23%, *t*_(31)_ = 4.016, *p* < 0.001; S→N: *M* = 55.47%, *t*_(31)_ = 2.634, *p* = 0.013). Generalization from nodding to shaking (only) was found in MT (*M* = 55.27%, *t*_(31)_ = 3.044, *p* = 0.005). Cross-classification accuracies, either from nodding to shaking or shaking to nodding, did not survive FDR correction in other ROIs (N→S: all *M*s < 55%, all *p*s > 0.03; S→N: all *M*s < 55%, all *p*s > 0.03). For completeness, we also conducted a two-way cross-classification analysis testing both directions (N→S, S→N) simultaneously (Fig. 4). This analysis revealed significant above-chance cross-classification (all *t*s > 2.4, all *p*s < 0.02) in all ROIs except for rSTS-F and rSTS-I (both *t*s < 2.1, both *p*s > 0.05).

#### Univariate analyses

**Figure 5.**
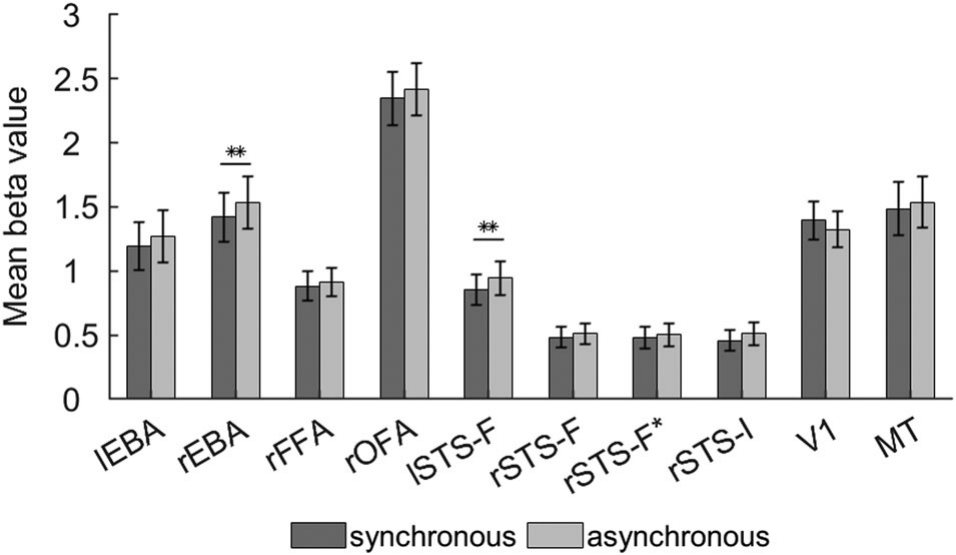
Mean *β* values for synchronous and asynchronous movement in each ROI. Error bars show standard error. The asterisks indicate above-chance accuracy after FDR correction for 10 comparisons: **p ≤ 0.01.

To compare univariate responses to synchronous and asynchronous movement, for each ROI and each participant we calculated the mean *β* value across all voxels included in the ROI, separately for synchronous and asynchronous stimuli (Fig. 5). We found significantly greater responses to asynchronous stimuli compared with synchronous stimuli in rEBA (*t*_(31)_ = 3.197, *p* = 0.003) and lSTS-F (*t*_(29)_ = 3.088, *p* = 0.004). Comparisons in other ROIs did not survive FDR correction (all *p*s > 0.02).

#### The functional characteristics of the EBA ROIs

The EBA is thought to show little or no face selectivity (Peelen and Downing, 2007). The ability of EBA to decode synchronous versus asynchronous head motion might therefore strike some readers as counterintuitive. With this in mind, we sought to confirm that our functionally defined EBA ROIs behaved as expected during the functional localizer procedure. During this procedure, participants viewed dynamic faces, bodies, and objects. Univariate contrasts were used to define face-selective (faces > objects) and body-selective (bodies > objects) regions. To examine whether our EBA ROIs showed unexpected face selectivity, we extracted *β* values for faces and objects in rEBA and lEBA and calculated the mean *β* value across all voxels included in each ROI, separately for faces and objects. Responses to faces and objects were similar in rEBA (*M*_faces_ = 2.346, *M*_objects_ = 2.481, *t*_(31)_ = 1.069, *p* = 0.293) and significantly greater for objects in lEBA (*M*_faces_ = 1.596, *M*_objects_ = 1.854, *t*_(31)_ = 2.749, *p* = 0.010). This pattern indicates that our functionally defined EBA ROIs behaved as expected; that is, the EBA ROIs showed no selectivity for faces per se.

### Exploratory whole-brain analyses

**Figure 6.**
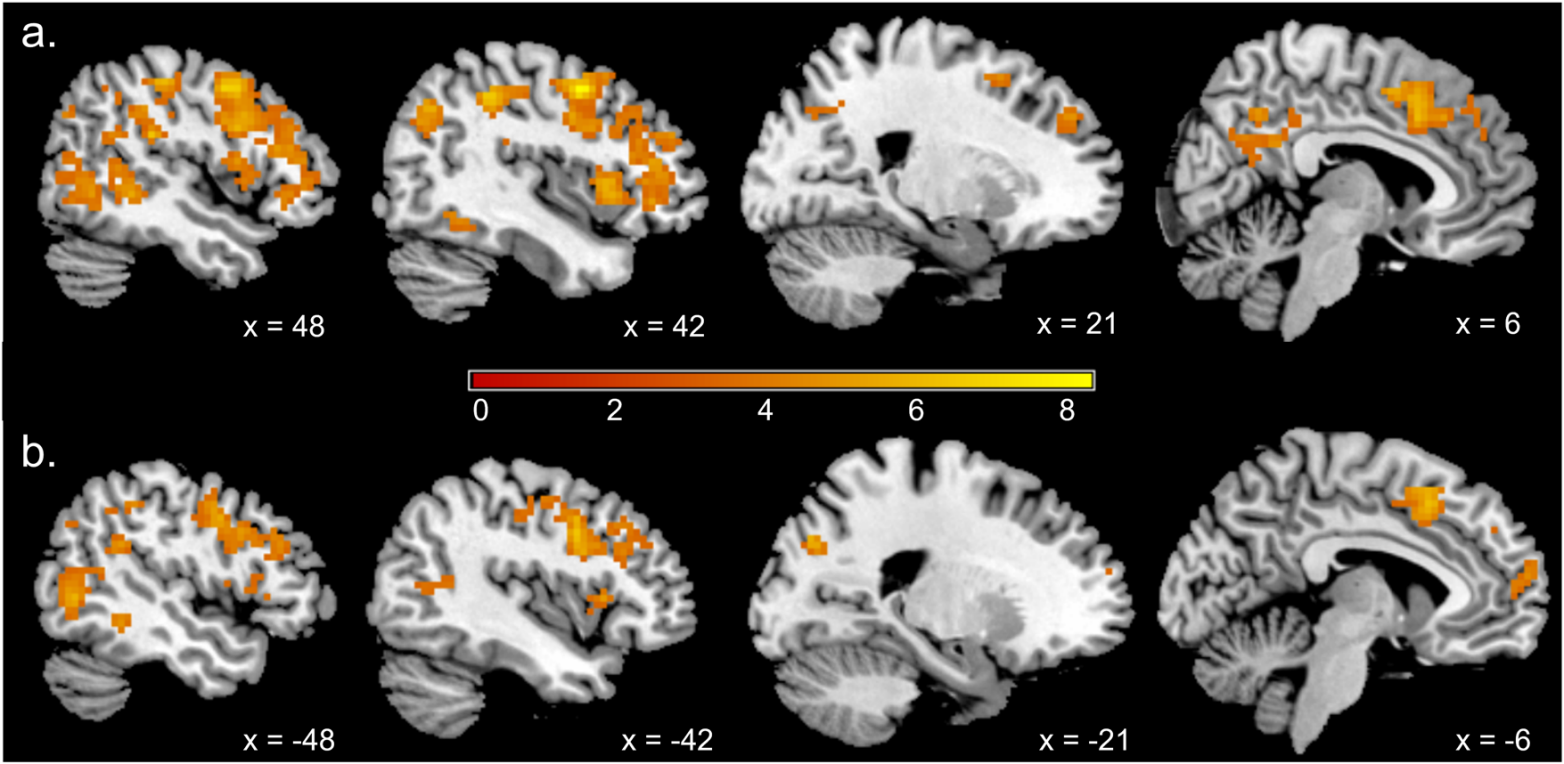
Results of whole-brain searchlight analysis: ***a***, right hemisphere; ***b***, left hemisphere.

**Figure 7.**
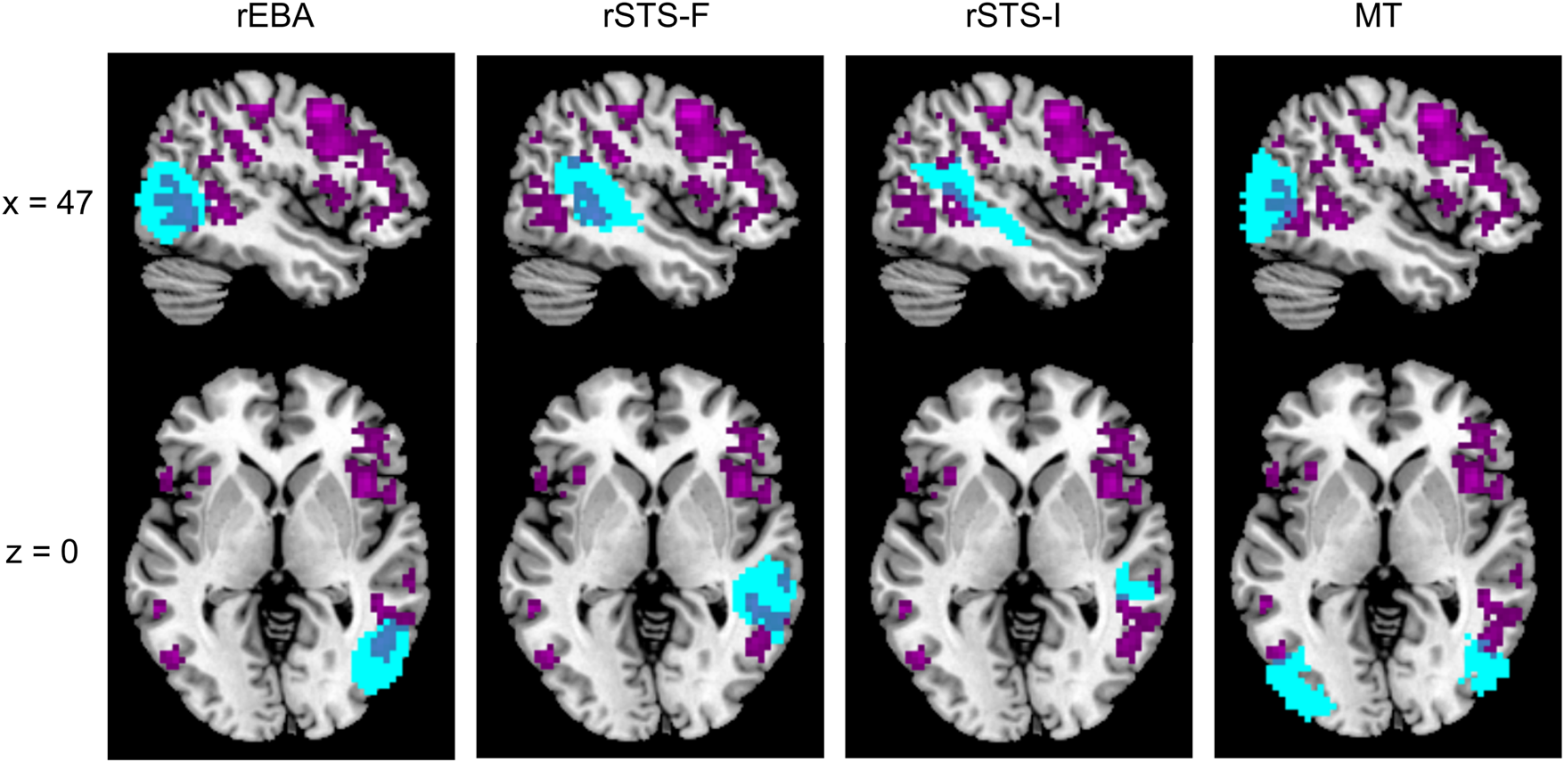
Overlap between the results of the whole-brain searchlight analysis (purple) and the group-level masks (cyan) that were used to define subject-specific ROIs for rEBA, rSTS-F, rSTS-I, and MT.

Whole-brain searchlight analysis identified multiple voxel clusters that supported the above-chance classification of synchrony versus asynchrony (Table 2; Fig. 6). The analysis revealed two large clusters with peak voxels in left (630 voxels) and right (1,401 voxels) precentral gyrus. The peak voxel of a third large cluster (717 voxels) was undefined by AAL but the largest contributing AAL region was the right supramarginal gyrus. Further clusters were identified with peak voxels in the left middle temporal gyrus (464 voxels), right precuneus (79 voxels), right angular gyrus (51 voxels), and left superior occipital gyrus (49 voxels). As expected, there was broad agreement between the results of the ROI and searchlight analyses; that is, those visual areas that supported significant levels of decoding in the ROI analyses were also identified by the searchlight analysis (Fig. 7).

**Table 2.** Results of whole-brain searchlight analysis

Hemisphere	Peak voxel location	Peak voxel				Cluster	
		MNI coordinates				Size	*p*-value
		*x*	*y*	*z*	*t*-value		
Right	Precentral gyrus	42	2	47	8.3701	1,401	<0.001
	Supramarginal gyrus^a^	33	−37	47	7.0992	717	<0.001
	Precuneus	6	−55	41	4.9307	79	<0.001
	Angular gyrus	42	−64	38	5.7106	51	0.001
	Cuneus	15	−70	35	5.2447	33	0.011
Left	Precentral gyrus	−42	5	35	6.4343	630	<0.001
	Middle temporal gyrus	−48	−64	2	5.8787	464	<0.001
	Superior occipital gyrus	−21	−76	35	5.7624	49	0.001
	Superior frontal gyrus, medial	−9	62	17	5.8307	43	0.003
	Left inferior parietal	−33	−46	41	4.8783	26	0.032

a The peak voxel of this cluster was in a region undefined by AAL, so the largest contributing AAL region is reported instead.

**Figure 8.**
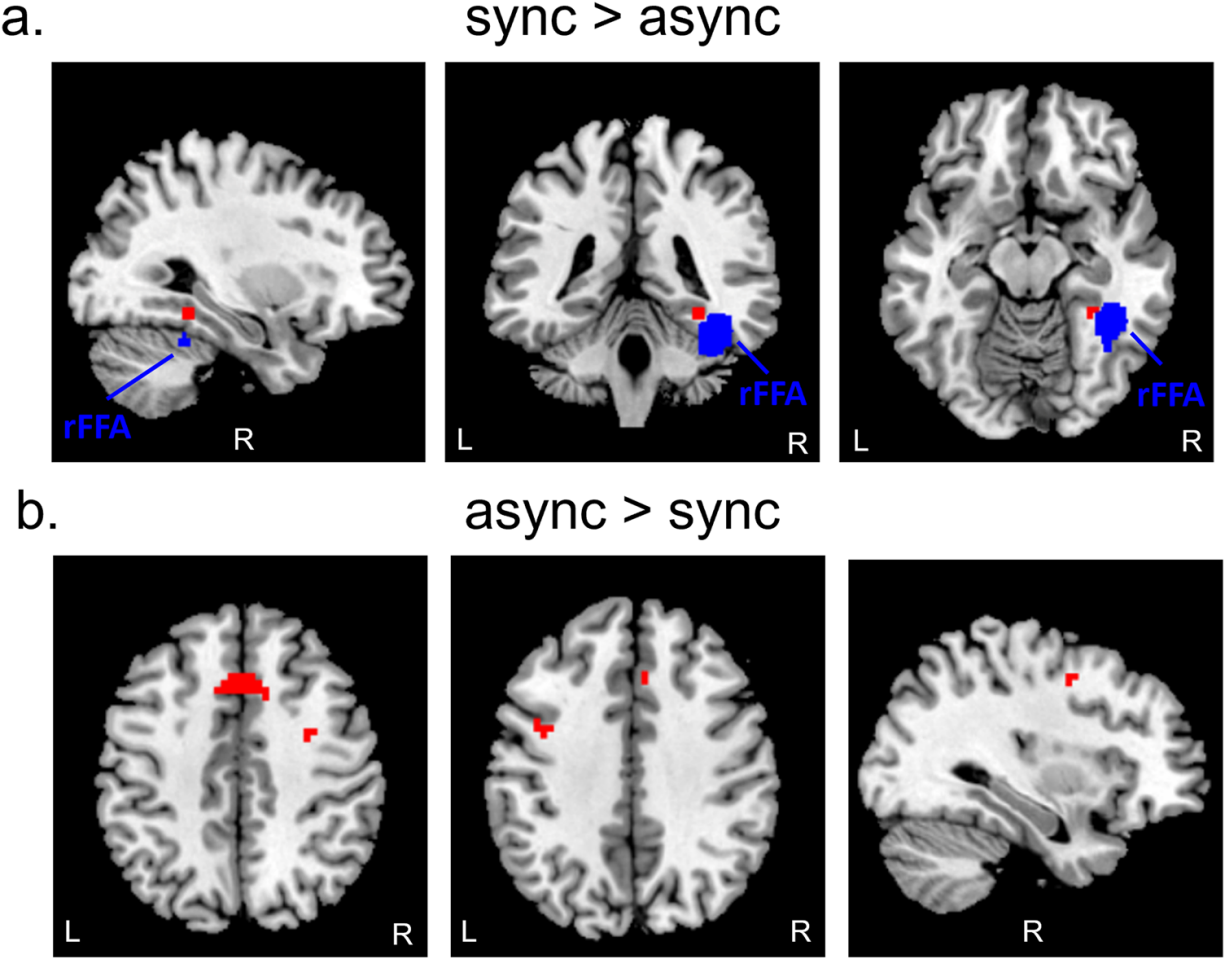
***a***, Whole-brain univariate analyses testing for regions showing greater responses to synchronous than asynchronous motion revealed a small cluster (red) with a peak in the right fusiform gyrus (*x* = 30, *y* = −40, *z* = −13). There was no overlap with the probabilistic map of rFFA (blue) that was used to define our individual FFA ROIs. ***b***, We also identified three clusters that responded more strongly to asynchronous than to synchronous motion with peaks in the left supplementary motor area (*k* = 65, *x* = −3, *y* = 17, *z* = 44) and left precentral gyrus (*k* = 7, *x* = −39, *y* = 2, *z* = 38) and within the right frontal lobe (*k* = 6, *x* = 33, *y* = −1, *z* = 44).

We also performed whole-brain univariate analyses to test for regions showing greater responses to synchronous than asynchronous motion, and vice versa (Fig. 8).

The contrast synchronous > asynchronous revealed a small cluster with a peak in the right fusiform gyrus (*k* = 7, *x* = 30, *y* = −40, *z* = −13, *t* = 7.020, *p* < 0.001). The contrast asynchronous > synchronous revealed three clusters with peaks in the left supplementary motor area (*k* = 65, *x* = −3, *y* = 17, *z* = 44, *t* = 8.874, *p* < 0.001) and left precentral gyrus (*k* = 7, *x* = −40, *y* = 2, *z* = 39, *t* = 6.591, *p* < 0.001) and within the right frontal lobe (peak region undefined; *k* = 6, *x* = 32, *y* = −1, *z* = 46, *t* = 6.601, *p* < 0.001).

It is possible that the activation in the right fusiform cortex seen for the contrast synchronous > asynchronous is attributable to rFFA. Note, however, that this univariate effect was not seen in the functionally defined FFA ROIs (Fig. 5). To further interrogate this possibility, we examined whether the cluster identified in the right fusiform cortex overlapped with the rFFA parcel described by Julian et al. (2012). We observed no overlap—the right fusiform cluster seen in our data was more medial than the rFFA parcel, adjacent to the parahippocampal gyrus.

## General discussion

The present study employed MVPA to identify visual regions that underlie the perception of interpersonal synchrony/asynchrony. Several regions of the social perception network were found to support significant decoding of synchronous versus asynchronous head movements, including bilateral EBA, face-selective and interaction-sensitive regions of the mid/posterior rSTS, and rOFA. We saw robust cross-classification in lEBA and rEBA, whereby a classifier trained to discriminate synchronous versus asynchronous head-shaking could also discriminate synchronous versus asynchronous head-nodding, and vice versa. Exploratory univariate analyses also identified a region of the right fusiform cortex that responded more strongly to synchronous than asynchronous motion.

There is growing speculation that social interactions may engage specialized visual processing beyond that recruited by the faces and bodies of the constituent individuals (Quadflieg and Koldewyn, 2017; Papeo, 2020; McMahon and Isik, 2023). Critically, the representation of relative-phase requires the extraction and integration of dynamic information from more than one person. Because synchrony/asynchrony is an emergent property of the kinematics of multiple actors, its representation cannot be attributed to augmented or additive processing of individuals. Our findings therefore provide important new evidence of multiactor visual processing within the social perception network.

### Contribution of EBA, pSTS, and OFA

Representations of interpersonal synchrony/asynchrony were found in bilateral EBA and in interaction-sensitive (STS-I) and face-selective (STS-F) regions of rSTS. Together with previous findings (Isik et al., 2017; Walbrin et al., 2018; Walbrin and Koldewyn, 2019; Abassi and Papeo, 2020, 2022; Bellot et al., 2021), these results suggest that EBA and pSTS may form part of a circuit that underlies the visual perception of social interactions. Previously, STS-I has been found to be more sensitive to social interactions than the adjacent and partially overlapping STS-F region (Isik et al., 2017). In our study, however, significant synchrony classification was seen in both regions.

The emerging body of evidence does not afford a straightforward account of the respective contributions of EBA and pSTS to social interaction perception. Despite its functional selectivity for body stimuli, EBA is thought to show little or no face selectivity (Peelen and Downing, 2007). Thus, one possibility is that the contribution of EBA to interaction perception is limited to the processing of body cues. At first glance, our findings—obtained with head and face stimuli—argue against this view. However, the synchronous and asynchronous stimuli used here depicted rigid head movements (nodding or shaking), rather than facial motion per se (O'Toole et al., 2002). While our stimuli are not canonical “body” stimuli, nor are they canonical “face” stimuli.

A second possibility is that EBA encodes spatial/temporospatial dyadic features (Quadflieg et al., 2015), whereas pSTS supports further interpretative processing (Centelles et al., 2011). This view is consistent with evidence that EBA is sensitive to the arrangement of actors within dyads (Abassi and Papeo, 2020, 2022), as well as the congruence (Quadflieg et al., 2015) and synchrony (current study) of dyadic interactions. Similarly, this account would explain why STS-I—but not EBA—exhibits sensitivity to interactive cues presented in the auditory domain (Landsiedel and Koldewyn, 2023). Moreover, this view potentially accords with evidence that different types of interaction (e.g., helping vs hindering) between simple moving shapes (e.g., circles and squares) can be decoded from the responses of STS-I (Isik et al., 2017; Walbrin et al., 2018). Given that the interactions depicted are between simple shapes, these results imply that STS-I represents relatively abstract features of social interactions, that is, that processing in this region is largely insensitive to the appearance and precise kinematics of the actors. To further interrogate the respective contributions of EBA and STS-I, it would be useful to establish whether the responses seen in EBA also support the classification of interactions between abstract geometric shapes.

We observed a notable difference between EBA and STS-I in our cross-classification analyses. Representations of synchrony/asynchrony generalized across motion types (nodding and shaking) in bilateral EBA. Interestingly, however, cross-classification did not exceed chance in STS-I. We speculate that responses in STS-I did not support significant cross-classification because head-shaking and head-nodding afford different social interpretations—disagreement and agreement. It is possible that the presence of synchrony affects perceived agreement and disagreement—abstract features potentially encoded within STS-I—but does so differently for nodding and shaking movements.

Univariate analyses of the responses seen in our ROIs revealed that rEBA and lSTS-F responded more strongly to asynchronous head movements than to synchronous head movements. This result mirrors a similar finding reported by Quadflieg et al. (2015) who observed greater activation in EBA and pSTS when participants viewed semantically incongruent face-to-face dyads, relative to semantically congruent dyads. Facing dyads may engage multiactor processing within the social perception network irrespective of interpersonal synchrony or asynchrony and semantic congruence or incongruence. However, asynchronous and incongruent dyads may be processed less efficiently than synchronous and incongruent dyads because they violate perceptual expectations about the likely appearance and kinematics of interactions.

Synchronous movement (Lakens, 2010; Lakens and Stel, 2011) and face-to-face arrangement (Papeo, 2020) are both thought to afford perceptual grouping whereby individual actors are processed as a single configuration. Interestingly, face-to-face (vs back-to-back) dyadic arrangements elicit stronger BOLD responses in EBA (Abassi and Papeo, 2020, 2022). Here, however, we observed stronger responses in EBA when participants viewed asynchronous (vs synchronous) movement. Studying how these univariate effects behave when dyadic synchrony (synchronous vs asynchronous movement) and arrangement (face-to-face vs back-to-back) are subject to factorial manipulation, may shed light on these somewhat contradictory results.

Significant synchrony/asynchrony classification in rOFA is unexpected given that previous manipulations of dyadic congruence (Quadflieg et al., 2015) and arrangement (Abassi and Papeo, 2020) failed to modulate neural responses in OFA. Relative to the stimuli used in previous studies, however, our stimuli emphasized the head and face regions. It is thus possible that OFA contributes to the perception of interpersonal synchrony by encoding the relative facial rotation and orientation of the different faces. This feature of our stimuli may also explain the robust synchrony/asynchrony decoding seen in STS-F. Unlike EBA, OFA did not support robust cross-classification.

EBA, pSTS, and OFA are key hubs within the social perception network that were identified in our study using functional localizers. The defining feature of each region is selectivity for, or sensitivity to, a particular type of social stimulus (e.g., bodies, faces, dyadic interactions). Our findings indicate that these regions contribute to the perception and representation of interpersonal synchrony. Further work is required to elucidate the specificity of this contribution, for example, whether the decoding effects described here are also seen when participants view synchronous and asynchronous interactions between pairs of nonfacing actors or between people and objects.

### Contribution of other areas

Significant decoding of synchrony/asynchrony in MT is consistent with evidence that MT responds more strongly to videos of dyadic interaction than to videos of people acting independently (Landsiedel et al., 2022) and more strongly to semantically incongruent dyads than to congruent dyads (Quadflieg et al., 2015). Given these findings, it is conceivable that MT contributes to the perception of dyadic interactions. However, a clear interpretation of the role of MT is complicated by the spatial overlap between MT and EBA (Ferri et al., 2013). In our study, the decoding of synchrony/asynchrony seen in MT did not survive the removal of overlapping EBA voxels. In contrast, the decoding seen in EBA remained significant when overlapping MT voxels were removed. Interpretation of these findings is further complicated by the fact that the MT region in our study was defined using a probabilistic group map approach, which, unlike a functional localizer approach, does not consider individual variation in the location of MT.

Whole-brain univariate analyses identified an area of the right fusiform cortex that showed selectivity for synchronous dyadic motion. Interestingly, there was no sign of this effect in the functionally defined rFFA ROI, nor was there any overlap with the probabilistic map of rFFA derived by Julian et al. (2012). Relative to interpersonal asynchrony, interpersonal synchrony affords grouping (Lakens, 2010; Lakens and Stel, 2011) and attributions of rapport (Miles et al., 2009; Lakens and Stel, 2011). One possibility is that this right fusiform region mediates additional processing engaged selectively by synchronous interactions. This exciting possibility warrants further investigation.

## Conclusion

Interpersonal synchrony is a critical cue when appraising dynamic social scenes. However, little is known about its neural representation within the human visual system (Cracco et al., 2022). Findings obtained with MVPA indicate that distributed responses throughout the social perception network support categorization of synchronous versus asynchronous head movements. Crucially, relative-phase is an emergent property of the actions of multiple actors. These results therefore provide important new evidence of multiactor visual processing within the social perception network.
